# A comparison of psychometric properties of two common measures of caregiving burden: the family burden interview schedule (FBIS-24) and the Zarit caregiver burden interview (ZBI-22)

**DOI:** 10.1186/s12955-020-01335-x

**Published:** 2020-04-06

**Authors:** Yu Yu, Zi-wei Liu, Tong-xin Li, Wei Zhou, Shi-jun Xi, Shui-yuan Xiao, Jacob Kraemer Tebes

**Affiliations:** 1 Hospital Evaluation Office, Xiangya Hospital, Central South University, Xiangya Road 87, Changsha, Hunan 410008 China; 2 Division of Prevention and Community Research, Department of Psychiatry, Yale School of Medicine, 389 Whitney Avenue, New Haven, CT 06511 USA; 3 School of Medicine, Hunan Normal University, 371 Tongzhi Road, Changsha, Hunan 410013 China; 4 Department of Social Medicine and Health Management, Xiangya School of Public Health, Central South University, Upper Mayuanlin Road 238, Changsha, Hunan 410008 China; 5 Hospital Administration Institute, Xiangya Hospital, Central South University, Xiangya Road 87, Changsha, Hunan 410008 China; 6 Mental Health Center, Xiangya Hospital, Central South University, Xiangya Road 87, Changsha, Hunan 410008 China

**Keywords:** Psychometric testing, FBIS, ZBI, Schizophrenia, Caregiver burden

## Abstract

**Purpose:**

The Family Burden Interview Schedule (FBIS-24) and the Zarit Caregiver Burden Interview (ZBI-22) are among the most widely used measures for assessing caregiving burden, but their psychometric performances have not been compared in the same study of caregivers of people living with schizophrenia (PLS). This is important because the measures assess overlapping constructs- the FBIS-24 assesses objective burden (e.g., completion of manual tasks) and the ZBI-22 assesses subjective burden (e.g., perceived distress, stigma). This study seeks to fill this gap by comparing the reliability and validity of the FBIS-24 and the ZBI-22 in a Chinese community sample of caregivers of PLS.

**Methods:**

A Cross-sectional stud was conducted in a community-based mental health service program in Central South part of China. A total of 327 primary family caregivers of PLS completed face-to-face interviews of the FBIS-24, the ZBI-22, the Patient Health Questionnaire (PHQ-9), the Generalized Anxiety Disorder Scale (GAD-7), and the Family Adaptation, Partnership, Growth, Affection and Resolve Index scale (APGAR), and PLS were assessed using the Global Assessment of Function scale (GAF).

**Results:**

Our findings show that both the FBIS-24 and ZBI-22 have comparable psychometric performance in terms of the internal consistency, convergent validity and known group’s validity.

**Conclusion:**

Both the FBIS-24 and the ZBI-22 are psychometrically sound measures of caregiving burden but the choice of which measure to use will depend on the research question.

## Background

In a context of de-institutionalization and community-based mental health services, the family caregiver’s role is becoming increasingly important at a sociological, economic, and political level [1, 2]. Caregivers serve as main providers of care for people with mental illness and thus shoulder a substantial part of the burden [3]. The burden of caregiving is often described as consisting of two parts: objective burden (such as completion of manual tasks and household duties) and subjective burden (such as caregiver perceptions of emotional distress and stigma) [4–6]. In combination, objective and subjective burden encompass physical, mental, financial and social aspects of caregiving [4]. Identifying a measure to assess burden for caregivers of people living with schizophrenia (PLS) is not only a vital step in understanding the types of support that caregivers need, but also critical to developing effective intervention programs that target specific caregiver needs [7, 8]. Although a plethora of measures have been developed to assess caregiving burden, two have been validated in international studies and used most often among caregivers of PLS: The Family Burden Interview Schedule (FBIS) [9] and the Zarit Caregiver Burden Interview (ZBI) [10].

The Family Burden Interview Schedule (FBIS) was initially developed by Pai and Kapur among Indian caregivers of psychiatric patients [9]. The original FBIS contains 26 items, with the first 24 items grouped under 6 categories to measure objective burden: financial burden, disruption of routine family activities, disruption of family leisure, disruption of family interaction, effect on physical health of others, and effect on mental health of others. Each item of objective burden is rated on a 3-point Likert scale (0 = no burden, 1 = moderate burden, 2 = severe burden). The 25th item comes as a supplementary question asking about any other family burden not mentioned in the first 24 items, and the 26th item serves as a subjective burden assessment by asking one standard question “How much would you say you have suffered owing to the patient’s illness?” on a 3-point Likert scale (0 = not at all, 1 = a little, 2 = severely). Most studies only use the first 24 items to assess objective family burden for convenient calculation and easy interpretation, which we refer to as FBIS-24. The total score of the FBIS-24 ranges from 0 to 48, with higher scores indicating higher objective burden. A recent study showed an optimal cut-off score of 23 to distinguish lower and higher burden for risk of psychological distress, with sensitivity being 76% and specificity being 68% for depression as measured by PHQ-9 [11]. The FBIS-24 has now been used among caregivers of a wide variety of health conditions as a standardized measure for assessing caregiving burden of both hospitalized and community living care-recipients [12–14].

The Zarit Caregiver Burden Interview (ZBI) was initially developed by Zarit and his colleagues among US caregivers of dementia seniors [15]. The original ZBI contains 29 items on a four-point Likert scale, which was later revised as 22 items on a five-point Likert scale and commonly referred to as ZBI-22 [10]. Each item of the ZBI-22 assess the respondent’s subjective burden by asking “do you feel or do you wish….” with optional answers scored from 0 to 4 (0 = never, 1 = rarely, 2 = sometimes, 3 = quite frequently, 4 = nearly always) except for the item 22 (0 = not at all, 1 = a little, 2 = moderately, 3 = quite a bit, 4 = extremely). The total score of the ZBI-22 ranges from 0 to 88, with higher scores indicating higher subjective burden [16]. A recent study showed an optimal cut-off score of 48 to distinguish lower and higher burden for risk of psychological distress, with sensitivity being 73% and specificity being 80% for depression as measured by PHQ-9 [17]. The ZBI-22 is the most widely used tool for measuring the level of subjective burden among caregivers, has been validated across many populations of caregivers (i.e. spouses/partners, children, and parents) and care-recipients (i.e. AD/dementia, physical illness, and mental illness), and is available in most languages (e.g., French, Spanish, and Chinese) [18, 19].

Although both measures have been used extensively in international studies to assess caregiving burden, data for the FBIS-24 and ZBI-22 have not been reported in the same study. A careful comparison between the two scales have shown that apart from differences in item number, Likert scale rating, score range, cut-off value, sensitivity, specificity and dimensionality, they also differ in their contents and emphasis (as shown in Table 1). FBIS-24 focuses on objective burden on the family level, while ZBI-22 targets at measuring subjective burden on individual level. Yet there is lack of evidence on the psychometric performance comparison between the two scales for burden assessment in the same study. The purpose of the present study is to address this issue by reporting for each measure: 1) sociodemographic and clinical differences for lower and higher levels of burden, 2) reliability (Cronbach’s alpha), and 3) validity in terms of convergent validity, and known groups’ validity for lower and higher levels of burden. Our sample is a Chinese rural community sample of caregivers of PLS in which all measures were collected simultaneously, thus allowing for the examination of unique and overlapping characteristics of objective and subjective burden using the FBIS-24 and ZBI-22, respectively.

**Table 1:** Simple comparison between FBIS-24 and ZBI-22

Characteristics	FBIS-24	ZBI-22
Item number	24	22
Likert scale rating	3-point (0-no,1-moderate, 2-serious)	5-point (0-never, 1-seldom,2-sometimes, 3-often, 4-always)
Score range	0–48	0–88
Cut-off value^&^	23 [11]	48 [17]
Sensitivity^&^	76%	73%
Specificity^&^	68%	80%
Dimensionality	6 (financial burden, disruption of routine family activities, family leisure, family interactions, and effect on physical and mental health of family members)	1–5 (different studies proposed different factors)

^&^calculation of cut-off value, sensitivity and specificity was based on two recent published papers on determining cut-off values for FBIS-24 and ZBI-22 to predict psychological distress including depression and anxiety. Here we only listed sensitivity and specificity based on the depression score as measured by PHQ-9

## Methods

### Participants and procedure

The study design and respondent recruitment have been described previously [20]. In brief, this is a cross-sectional study using a one-stage cluster sampling in Ningxiang County of Hunan province between November 2015 and January 2016. Three towns and 1 xiang were randomly selected as the study districts, which includes 55 communities/villages as our sampling frame. Participants were recruited through a community-based mental health service program named “686 Program”, which is China’s largest demonstration project in mental health service aimed at integrating hospital and community services for serious mental illness [21]. In each community/village, one primary family caregiver of every PLS registered in the “686 Program” was enrolled as the target population. The inclusion criterion include: 1) PLS being registered in 686 Program; 2) PLS fulfilling the Chinese Classification of Mental Disorders-3 (CCMD-3) or the International Classification of Diseases-10 (ICD-10) criteria for schizophrenia; 3) the primary caregiver is living with the PLS and has taken the most responsibility of caring, with full understanding about the situation of both the PLS and the family; 4) primary caregiver older than 16 years old; 5) primary caregiver is able to understand and communicate. The exclusion criteria include: 1) PLS with diagnosis other than schizophrenia such as depression and epilepsy; 2) PLS living alone; 3) primary family caregivers having serious physical or mental illness that are unable to communicate. Three hundred fifty-two primary family caregivers of PLS were selected as the final sample.

The study received ethics committee approval from the Xiangya School of Public Health of Central South University. The researchers, accompanied by community/village doctors went door-to-door to each participant’s home, explained the study to the participants, and then obtained written informed consent. Participants were invited to complete questionnaires to assess their caregiving experiences and their psychological well-being in face-to-face interviews. Each interview lasted for about 40–50 min, and participants were reimbursed with some small gifts equivalent to RMB ¥ 10 (equal to USD$1.4) in return for their participation. Among the 352 family caregivers we approached, 327 agreed to participate the study and completed the interview (response rate 93%). Among the 327 respondents, 292 completed all questionnaires with no missing data on all study measures, and 295 completed both the FBIS-24 and ZBI-22 with no missing data. The sample size satisfies the requirement of at least 5 participants for each item of the scale to conduct a psychometric testing [22].

### Measures

### Family burden interview schedule (FBIS-24)

The FBIS-24 [9] consists of 24 items rated on a 3-point Likert scale from 0 (no burden) to 2 (serious burden). The total score ranges from 0 to 48 with higher scores showing higher burden. The FBIS-24 has been widely used and well validated in numerous previous studies, with a Cronbach’s α of 0.90 in the original scale [9, 12, 23]. In the current study, the Chinese version of FBIS showed acceptable internal consistency with a Cronbach’s α of 0.86.

### Zarit burden interview (ZBI-22)

The ZBI-22 consists of 22 items scored on a 5-point Likert scale from 0 (never) to 4 (nearly always), except for the final item on global burden, rated from 0 (not at all) to 4 (extremely). The total score ranges from 0 to 88 with higher scores indicating higher burden. The ZBI-22 has been widely used and well validated in numerous previous studies, with a Cronbach’s α of 0.92 in the original scale [24–26]. In the current study, the Chinese version of ZBI showed acceptable internal consistency with a Cronbach’s α of 0.89.

### Patient health questionnaire (PHQ-9)

The PHQ-9 [27] consists of 9 items rated on a 4-point Likert scale from 0 (not at all) to 3 (nearly every day). The total score ranges from 0 to 27, with a cut-off point of 10 differentiating depression and non-depression [28]. The PHQ-9 has been widely used and well validated in numerous previous studies, with a Cronbach’s α of 0.89 in the original scale [27–30]. In the current study, the Chinese version of the PHQ-9 demonstrated good internal consistency with a Cronbach’s α coefficient of 0.89.

### Generalized anxiety disorder scale (GAD-7)

The GAD-7 [31] consists of 7 items rated on a 4-point Likert scale from 0 (not at all) to 3 (nearly every day). The total score ranges from 0 to 21, with a cut-off point of 10 differentiating anxiety and non-anxiety [32]. The GAD-7 has been widely used and well validated in numerous previous studies, with a Cronbach’s α of 0.92 in the original scale [31, 33]. In the current study, the Chinese version of the GAD-7 demonstrated good internal consistency with a Cronbach’s α coefficient of 0.91.

### Family adaptation, partnership, growth, affection and resolve index scale (APGAR)

The APGAR [34] consists of 5 items scored in 3-point Likert scale from 0 (hardly ever) to 2 (almost always). The total score ranges from 0 to10 with higher scores indicating higher satisfaction with family functioning. The APGAR has been widely used and well validated in numerous previous studies, with a Cronbach’s α of 0.86 in the original scale [35–37]. In the current study, the Chinese version of APGAR showed good internal consistency with a Cronbach’s α coefficient of 0.91.

### Global assessment of functioning (GAF)

The GAF was used to assess the PLS’s overall functioning and consists of one 100-point single item covering three major domains: social functioning, occupational functioning, and psychological functioning [38]. The total score ranges from 1 to 100, with higher scores indicating higher overall functioning. Examples are given for each 10-level interval.

### Statistical analyses

All analyses were performed using the Statistical Package for Social Sciences 16.0 (SPSS Inc., Chicago, IL, USA). Data were expressed as mean ± SD, median (interquartile range) or number (%), as appropriate. Group differences were analyzed using the Student’s t-test or Mann–Whitney U test for continuous variables and Chi-square tests for categorical variables. Internal consistency was examined by calculating Cronbach’s alpha, with a recommended level of 0.8 and above indicating good internal consistency [39]. Convergent validity was measured using Spearman correlations with expected significant positive correlations with caregiver’s depression (PHQ-9 score) and anxiety symptoms (GAD-7 score), as well as significant negative correlations with family functioning (APGAR score) and PLS functioning (GAF score) [3, 40–43]. Known group’s validity was assessed using Mann-Whitney U tests, with expected higher burden scores among caregivers with a physical illness than those without [5, 20]. The response agreement between the FBIS-24 and the ZBI-22 was evaluated with Cohen’s kappa, with values< 0 indicating no agreement and 0–0.20 as slight, 0.21–0.40 as fair, 0.41–0.60 as moderate, 0.61–0.80 as substantial, and 0.81–1 as almost perfect agreement [44]. A significance value of *p* < 0.05 (2-tailed) was considered significant. According to Cohen [45], correlation coefficients below 0.30 were considered small, between 0.30–0.50 were considered medium, and above 0.50 were considered large. Among the 327 respondents we interviewed that completed all information on socio-demographics, 295 completed both FBIS-24 and ZBI-22 with no missing data, while 292 completed all questionnaires with no missing data. No significant difference was observed in socio-demographic information between respondents with no missing data and respondents with missing data, so we used the default listwise or pairwise deletion for missing values in the following analysis.

## Results

### Group comparisons of socio-demographic characteristics using two burden measures

Table 2 shows the socio-demographic characteristics of the sample and their comparisons between lower and higher burden groups. The median (interquartile range) age of the caregivers was 59 (49–67) years, most of them were married (82.26%) and with a primary grade education (59.94%). Slightly more than half of the caregivers were female (53.82%) and employed (52.91%). The two largest family relationships to the PLS were as a parent (46.18%) or spouse (34.56%). The caregivers spent a median (q1–q3) of 15 years (9–25) caregiving.

**Table 2:** Demographic characteristics of caregivers categorized by validated cutoff values of FBIS-24 and ZBI-22 (*n* = 327)

Variables		Total	FBIS ≤ 23	FBIS > 23	z/χ^**2**^	*P*	ZBI ≤ 48	ZBI > 48	z/χ^**2**^	*P*
Age (years)	median (IQR, min-max)	59 (49–67; 17–81)	59 (48–67; 17–81)	58 (50–66; 25–81)	0.345	0.730	59 (48–67; 17–81)	59 (50–66; 29–77)	0.103	0.918
Gender	Male	151 (46.18)	74 (52.48)	59 (38.06)	6.203	**0.013**	87 (48.60)	46 (38.33)	3.068	0.080
	Female	176 (53.82)	67 (47.52)	96 (61.94)			92 (51.40)	74 (61.67)		
Marriage*	Married	269 (82.26)	119 (84.40)	130 (83.87)	0.0153	0.902	153 (85.47)	98 (81.67)	0.773	0.379
	Unmarried	58 (17.74)	22 (15.60)	25 (16.13)			26 (14.53)	22 (18.33)		
Occupation	Employed	173 (52.91)	78 (55.32)	78 (50.32)	0.740	0.390	93 (51.96)	64 (53.33)	0.0547	0.815
	Unemployed	154 (47.09)	63 (44.68)	77 (49.68)			86 (48.04)	56 (46.67)		
Education	Primary	196 (59.94)	82 (58.16)	93 (60.00)	6.320	**0.042**	98 (54.75)	78 (65.00)	7.411	**0.025**
	Middle	87 (26.61)	32 (22.70)	47 (30.32)			48 (26.82)	33 (27.50)		
	High	44 (13.46)	27 (19.15)	15 (9.68)			33 (18.44)	9 (7.50)		
Kinship	Spouses	113 (34.56)	54 (38.30)	45 (29.03)	9.794	**0.007**	66 (36.87)	34 (28.33)	12.917	**0.002**
	Parents	151 (46.18)	52 (36.88)	85 (54.84)			68 (37.99)	70 (58.33)		
	Other	63 (19.27)	35 (24.82)	25 (16.13)			45 (25.14)	16 (13.33)		
Care duration (years)		15 (9–25; 1–49)	16 (8–25, 1–49)	14 (9–23; 2–44)	0.630	0.529	15 (8–25; 1–49)	15 (9.5–23; 2–44)	−0.173	0.863

Note: numbers in bold means significant at *P* = 0.05

*Married includes married and cohabited, unmarried includes single, divorced, and widowed

FBIS-24: 24-item Family Burden Interview Schedule; ZBI-22: 22-item Zarit Burden Interview

Using a score of 23 and 48 as the cut-offs for FBIS-24 (objective burden) and ZBI-22 (subjective burden) to distinguish those with higher and lower burden, we compared these groups on demographic characteristics. Overall, the demographic characteristics of these two groups were comparable. Relative to the lower burden group, caregivers in the higher burden group were more likely to be parents (54.84% vs 36.88% for FBIS-24, 58.33% vs 37.99% for ZBI-22) and less likely to have a high school education (9.68% vs 19.15% for FBIS-24, 7.50% vs 18.44% for ZBI-22). For either measure, no significant differences were found between lower burden and higher burden groups based on caregiver age, marital status, occupation, and number of years caregiving. However, there were more female caregivers with higher objective burden (FBIS-24) than with lower burden (61.94% vs 47.52%, *p* < 0.05), but this gender difference was only observed at a trend level of significance for subjective burden with the ZBI-22 (61.67% vs 51.40%, *p* = 0.080).

### Group comparisons of clinical characteristics

Table 3 shows the clinical characteristics and comparisons between lower and higher burden groups for the two measures. The median score of depression, anxiety, and family functioning of caregivers as measured by PHQ-9, GAD-7, and APGAR were 9 (4–15), 9 (4–15) and 7 (4–9), respectively. The median score of PLS functioning as measured by GAF was 41 (20–61).

**Table 3:** Clinical characteristics of caregivers categorized by validated cutoff values of FBIS-24 and ZBI-22 (n = 292)

Variables	Total	FBIS ≤ 23	FBIS > 23	z/χ^**2**^	P	ZBI ≤ 48	ZBI > 48	z/χ^**2**^	P
Depression (PHQ-9)	9 (4–15; 0–27)	6 (2–9; 0–27)	14 (8–18.5; 0–27)	−8.30	**< 0.001**	6 (2–9; 0–26)	15 (10–20; 0–27)	−9.58	**< 0.001**
Anxiety (GAD-7)	9 (4–15; 0–21)	6 (1–9; 0–21)	13 (7–18; 0–21)	−7.99	**< 0.001**	6 (1–10; 0–21)	14 (9–18; 0–21)	−8.95	**< 0.001**
Family functioning (APGAR)	7 (4–9; 0–10)	8 (5–10;0–10)	6 (3–9; 0–10)	3.30	**0.001**	8 (5–10; 0–10)	5 (2–8; 0–10)	5.12	**< 0.001**
PLS functioning (GAF)	41 (20–61; 1–99)	55 (30–68;4–90)	30 (15–55; 1–99)	5.21	**< 0.001**	55 (30–65;4–99)	23 (13–50.5; 1–85)	6.45	**< 0.001**

Note: numbers in bold means significant at *P* = 0.01

FBIS-24: 24-item Family Burden Interview Schedule; ZBI-22: 22-item Zarit Burden Interview; PHQ-9: Patient Health Questionnaire-9; GAD-7: Generalized Anxiety Disorder Scale-7; APGAR: Family Adaptation, Partnership, Growth, Affection and Resolve Index scale; GAF: Global Assessment of Functioning

Clinical comparisons between higher and lower burden using cut-off scores of 23 and 48 for FBIS-24 and ZBI-22, respectively, showed comparable patterns between the two measures, regardless of whether the assessment was of objective or subjective burden. Relative to caregivers with lower burden, those with higher burden also reported higher levels of depression (14 vs 6 for FBIS-24, 15 vs 6 for ZBI-22) and anxiety (13 vs 6 for FBIS-24, 14 vs 6 for ZBI-22) scores, lower family functioning scores (6 vs 8 for FBIS-24, 5 vs 8 for ZBI-22), and lower PLS functioning (30 vs 55 for FBIS-24, 23 vs 55 for ZBI-22).

### Psychometric comparisons

Table 4 shows comparisons of reliability and validity results between the FBIS-24 and ZBI-22. Overall, reliability for each measure was generally good, with Cronbach’s alpha at 0.86 for the FBIS-24 and 0.89 for the ZBI-22. Convergent validity was also in general accordance with expectations and similar between the FBIS-24 and ZBI-22, with significant positive correlations with PHQ-9 score (*r* = 0.51 and 0.64, *p* < 0.01) and GAD-7 score (*r* = 0.49 and 0.56, *p* < 0.01), and significant negative correlations with APGAR score (*r* = − 0.26 and − 0.31, *p* < 0.01) and GAF score (*r* = − 0.43 and − 0.46, *p* < 0.01), respectively. All correlations were of medium to large effect size, except for the APGAR score, indicating family functioning may be less strongly correlated to caregiver burden than PLS functioning and caregiver psychological distress. For known groups’ validity, both the FBIS-24 and ZBI-22 showed significantly higher burden scores in caregivers with a physical illness than for those without a physical illness (26 vs 22 and 47 vs 36.5, *p* < 0.05).

**Table 4:** Reliability and Validity of the FBIS-24 and ZBI-22(*n* = 292)

Property	FBIS-24	ZBI-22
**Internal consistency**		
Cronbach’s α	0.864	0.888
**Convergent validity**		
Caregiver depression (PHQ-9)	0.505**	0.639**
Caregiver anxiety (GAD-7)	0.494**	0.558**
Family functioning (APGAR)	−0.261**	− 0.307**
PLS functioning (GAF)	−0.427**	− 0.455**
**Known groups’ validity**		
Caregivers with physical illness	26 (18–31; 0–44)	47 (33–56; 1–84)
Caregivers without physical illness	22 (12–29; 2–44)	36.5 (20–55.5; 0–88)
*Z*	−1.967	− 2.508
*P*	0.049	0.012
**Cohen’s kappa**	0.523	< 0.001

FBIS-24: 24-item Family Burden Interview Schedule; ZBI-22: 22-item Zarit Burden Interview; PHQ-9: Patient Health Questionnaire-9; GAD-7: Generalized Anxiety Disorder Scale-7; APGAR: Family Adaptation, Partnership, Growth, Affection and Resolve Index scale; GAF: Global Assessment of Functioning

Among the 295 caregivers with non-missing data in both FBIS-24 and ZBI-22, most of caregivers (52.54%) were identified as having higher family burden using the FBIS-24 cut-off of 23, whereas the prevalence of higher burden was 40.0% in the same group using the ZBI-22 cut-off of 48, with moderate chance-corrected agreement between the two instruments (Cohen’s kappa: 0.52). The overlap of higher burden prevalence is illustrated as following: 101 of the 295 caregivers (34.2%) were captured by both FBIS-24 > 23 and the ZBI-22 > 48.

## Discussion

This study is the first to compare the psychometric properties of two widely used measures of caregiving burden, the FBIS-24 and the ZBI-22, as indicators of objective and subjective burden for caregivers of PLS. We examine sociodemographic and clinical differences, reliability and validity for lower and higher burden scores on each measure among Chinese caregivers. Our findings indicate that both scales demonstrate acceptable psychometric performance with respect to the internal consistency, convergent validity and know group’s validity, and moderate chance-corrected agreement between them (Cohen’s kappa: 0.52).

What is most striking is how comparable both measures are psychometrically, despite measuring slightly different aspects of the same construct, caregiver burden. Both measures demonstrate strong internal consistency and are significantly associated with expected clinical indicators of caregiver depression, anxiety, and family functioning, and PLS functioning. In addition, both measures reveal a gender difference in caregiving burden in which women report significantly higher levels of objective burden on the FBIS-24 and higher subjective burden at a trend level of significance on the ZBI-22. Higher burden among women is consistent with gender role theory and is consistent with the caregiving literatures [46–48]. Some recent research has also noted that women may have fewer resources available to them for caregiving and use less effective ways of coping [49–52].

The comparable psychometric properties of the FBIS-24 and ZBI-22 indicate that the two scales may be used interchangeably in assessment of caregiver burden. The choice between FBIS-24 and ZBI-22 may depend on your research question and interest based on the observed differences such as sensitivity, specificity and contents, as shown in Table 1. For instance, FBIS-24 focuses on objective burden at family level, with higher sensitivity at cut-off value of 23 than ZBI-22 (76% vs 73%) [11]. Meanwhile, ZBI-22 mainly targets at subjective burden at individual level, with higher specificity at cut-off value of 48 than FBIS-24 (80% vs 68%) [17]. If the primary interest is addressing objective indicators of burden related to manual tasks and duties at family level, the FBIS-24 may be particularly useful in identifying a broad spectrum of caregivers for assessment or intervention purposes. In contrast, if the primary interest is excluding individuals for assessment or intervention with lower levels of subjective burden at personal level, in terms of emotional distress and experiences of stigma, then use of the ZBI-22 to do so may be more appropriate.

Another implication of the present findings is that the use of both measures in the same study, may be warranted as the FBIS-24 and ZBI-22 complement one another in our understanding of caregiver burden, and offer consistent correspondence to one another to related sociodemographic and clinical measures of a study population [53]. Although it may not be feasible to use both measures in real-world contexts, future research should examine ways that both measures offer a more precise and nuanced understanding of caregiver burden that facilitates assessment and subsequent intervention.

This study has a few limitations. First, the data reported is cross-sectional, and so no longitudinal psychometric data is reported for each measure. However, this limitation is mitigated somewhat because the purpose of this study was to examine psychometric properties of each measure and their relationship to various concurrent sociodemographic and clinical indicators. Second, the sample came from a single study population, a relatively rural county of Hunan province, which may limit the study’s representativeness. Future research should examine our findings in additional populations.

## Conclusions

In conclusion, the present study provides empirical support for the psychometric comparability of two measures of caregiver burden, the FBIS-24 and the ZBI-22, for the reliable and valid assessment of caregiver objective and subjective burden, respectively. The choice between the FBIS-24 and the ZBI-22 for assessment of caregiver burden depends on your research question and interest.

## Data Availability

The datasets used and/or analyzed during the current study are available from the corresponding author on reasonable request.
